# Risk factors of Shiga toxin-producing *Escherichia coli* in livestock raised on diversified small-scale farms in California

**DOI:** 10.1017/S0950268822001005

**Published:** 2022-06-01

**Authors:** Laura Patterson, Nora Navarro-Gonzalez, Michele T. Jay-Russell, Peiman Aminabadi, Alda F. A. Pires

**Affiliations:** 1 Department of Population Health and Reproduction, University of California-Davis, Davis, CA 95616, USA; 2 Western Center for Food Safety, University of California-Davis, Davis, CA 95616, USA

**Keywords:** Food safety, mixed crop–livestock farms, organic production, pasture-raised livestock, Shiga toxin-producing *Escherichia coli*, sustainably produced

## Abstract

The increasing number of diversified small-scale farms (DSSF) that raise outdoor-based livestock in the USA reflects growing consumer demand for sustainably produced food. Diversified farms are small scale and raise a combination of multiple livestock species and numerous produce varieties. This 2015–2016 cross-sectional study aimed to describe the unique characteristics of DSSF in California, estimate the prevalence of Shiga toxin-producing *Escherichia coli* (STEC) in livestock and evaluate the association between risk factors and the presence of STEC in livestock, using generalised linear mixed models. STEC prevalence was 13.62% (76/558). Significant variables in the mixed-effect logistic regression model included daily maximum temperature (OR 0.95; CI_95%_ 0.91–0.98), livestock sample source (cattle (OR 4.61; CI_95%_ 1.64–12.96) and sheep (OR 5.29; CI_95%_ 1.80–15.51)), multiple species sharing the same barn (OR 6.23; CI_95%_ 1.84–21.15) and livestock having contact with wild areas (OR 3.63; CI_95%_ 1.37–9.62). Identification of STEC serogroups of public health concern (e.g. O157:H7, O26, O103) in this study indicated the need for mitigation strategies to ensure food safety by evaluating risk factors and management practices that contribute to the spread and prevalence of foodborne pathogens in a pre-harvest environment on DSSF.

## Introduction

The increasing number of diversified small-scale farms (DSSF) that raise outdoor-based (i.e. grass-fed, pasture-raised) livestock in the USA reflects growing consumer demand for sustainably produced local foods, including animal products such as meat and eggs [1–5]. California is the top producer in the USA of agricultural products, as well as organic food sales, which includes products from DSSF [3, 6]. However, there is a lack of science-based information characterizing the risk factors associated with the prevalence of foodborne pathogens, such as Shiga toxin-producing *Escherichia coli* (STEC), in livestock raised on DSSF.

Diversified farms are most often small scale and can raise a combination of multiple livestock species and numerous produce varieties, with the intent of selling specialised (e.g. organically grown) animal products directly to consumers [5]. Some DSSF integrate livestock and crop production (i.e. integrated crop–livestock farms) by using their animals to graze crop residues or cover crops before planting a field with fresh produce [1, 7]. Grazing improves soil fertility and provides farm owners with another source of revenue through fibre or meat products [1, 8]. Additionally, many countries promote diversified farming systems as an alternative to intensive agriculture, displaying a willingness to transition to agroecology, in line with the UN Sustainable Development Goal number 12: ‘Ensure sustainable consumption and production patterns’ [9]. This goal may lead to an increase in the number of DSSF and products globally.

Many consumers perceive produce and meat raised on small-scale farms as more natural or safer (e.g. pesticide-free) than food grown on large-scale conventional farms or meat animals raised in confinement systems [2, 10]. However, livestock are asymptomatic reservoirs for foodborne pathogens, and without adequate mitigation strategies, these pathogenic microorganisms may enter the food supply [11–16]. Livestock are intermittent shedders of enteric pathogens and shedding may increase under certain conditions, such as during specific seasons or periods of stress (e.g. transportation), or due to certain management practices (e.g. stocking rate) [7, 17–19]. STEC can survive in the soil for extended periods of time and spread to humans directly through contact with livestock or indirectly via contaminated food (e.g. fresh produce, meat) or water [15, 17, 20–22]. STEC remains one of the main enteric pathogens associated with foodborne outbreaks in the USA [20, 21]. The top seven STEC O-serogroups that cause the most severe illness in humans include O157:H7, O26, O103, O111, O121, O145 and O45 [23]. Beef products and vegetables are common sources of infection [20,21]. However, outbreaks linked to organic animal products have been reported in small numbers [10]. Fresh produce consumed raw are considered high-risk crops, and when contaminated by livestock or wildlife faeces containing STEC, they can become a vehicle for these pathogens to enter the food supply [21,22, 24]. For instance, cattle grazing uphill from a produce field was likely the causative factor for the 2019 *E. coli* O157:H7 romaine lettuce outbreak [25].

The aim of this study was to (a) describe the unique characteristics of DSSF, (b) estimate the prevalence of STEC in livestock and (c) evaluate the association between risk factors and the presence of STEC in livestock raised on DSSF located in California.

## Methods

### Study design and farm enrolment

During 2015–2016, we conducted a repeated cross-sectional study in California to test for *Campylobacter* spp., *Salmonella* spp., non-O157 STEC and *E. coli* O157:H7 in faecal samples collected from livestock raised on DSSF [11]. Enrolment criteria for participating DSSF was based on the United States Department of Agriculture Economic Research Service (USDA-ERS) definition of small-scale farms [4], but adjusted to fit the unique growing conditions of California operations: (1) small- to medium-scale farm (i.e. poultry producers raising <1000 birds annually or livestock producers with an annual gross-sales <$500 000 and that raised a maximum of 500 goats/sheep and/or 100 cattle and/or 100 pigs); (2) raised a diversity of crops and/or multiple species of livestock and poultry; (3) marketed farm products directly to consumers (e.g. farmers markets, community-supported agriculture programs); and (4) willingness to participate. We enrolled farms from four regions of northern and central California that corresponded to the diversity of bioregions and that enabled an acceptable time between sample collection and laboratory processing [26]. Farm recruitment occurred through personal communications, announcements at agriculture outreach seminars and contact at farmers' markets or agricultural fairs.

### Sample collection

Farms were visited twice between May 2015 and June 2016, once during each of the following periods: summer/autumn or winter/spring, which reflect California's growing seasons and the seasonality of STEC shedding [11]. Livestock species sampled in this study included dairy and beef cattle, dairy and meat goats, pigs and sheep. Sample sizes were calculated using Epitools [27] based on the total number of animals on each farm, with an assumed STEC prevalence of 5% and 10% precision error, stratified by each livestock species. Individual fresh faecal samples were collected from the ground of barns or pastures. Samplers wore gloves and placed approximately 50–100 g of faeces into each sterile whirl-pak bag (Nasco, Modesto, CA, USA). Bags were immediately placed into plastic coolers containing ice packs, transported to the laboratory at the end of the sampling day and most samples were processed within 24 h.

### *Escherichia coli* O157:H7 and non-O157 STEC culture and PCR

STEC was isolated from faecal samples as described previously with modifications [28]. In brief, 10 g of faecal material was placed in 90 ml of Tryptic Soy Broth (TSB; Becton, Dickinson and Company, Franklin Lakes, NJ, USA). Samples were then incubated for 2 h at 25 °C with 100 rpm agitation, followed by 8 h at 42 °C with 100 rpm agitation and held overnight at 6 °C, using a Multitron programmable shaking incubator (Eppendorf, Hauppauge, NY, USA). For detecting *E. coli* O157:H7, immunomagnetic separation using Dynal anti-*E. coli* O157 beads (Invitrogen/Dynal, Carlsbad, CV, USA) was performed on TSB enrichment broths with the automated Dynal Bead Retriever (Invitrogen) per the manufacturer's instructions. After incubation and washing, 50 μl of the resuspended beads were plated onto Rainbow agar O157 (Biolog, Hayward, CA, USA) with novobiocin (20 mg/l) and tellurite (0.8 mg/l) (MP Biomedicals, Solon, OH, USA). Fifty microlitres of the resuspended beads were also plated onto MacConkey II Agar using sorbitol supplemented with potassium tellurite (2.5 mg/l) and Cefixime (0.05 mg/l) (CT-SMAC); plates were streaked for isolation and incubated for 24 h at 37 °C. Suspect *E. coli* O157:H7 isolates were confirmed using traditional PCR for the *rfbE* gene [29].

To detect non-O157 STEC, 1 ml of TSB enrichment broth was incubated in mEHEC selective media (Biocontrol, Bellevue, WA, USA) for 12 h at 42 °C followed by plating and incubating on Chromagar STEC (DRG International Inc., Springfield, NJ, USA) at 37 °C overnight. Up to eight presumptive STEC-positive colonies, demonstrating mauve colour and glowing under UV light, were confirmed for the presence of *stx1* and/or *stx2* genes by real-time PCR [28]. Confirmed STEC isolates were then characterised for virulence genes (*stx1*, *stx2*, *eaeA*, *hlyA* and *ehxA*) using conventional PCR [29]. After PCR testing, one isolate with a unique virulence gene profile from each positive sample was submitted to the Pennsylvania State University *E. coli* Reference Center to confirm O-serogroups [28, 30].

### Farmer questionnaire and environmental factors

A 41-question questionnaire, consisting of mainly closed-ended questions, was given to farm owners mostly in person. The questionnaire included sections regarding farm demographics, animal health and farm management practices. Variables analysed for model building included risk factors from the farmer questionnaire, sample day factors (e.g. temperature) and farm-level characteristics (e.g. whether a farm raised multiple types of livestock). Weather data from the nearest California Irrigation Management Information System weather station (http://www.cimis.water.ca.gov) within a similar microclimate provided environmental factors to be included in model building (e.g. average relative humidity, daily maximum temperature) [31]. Also, USDA plant hardiness zones, which are based on the average annual minimum winter temperature, were included as a proxy for the many microclimates in California [32]. Only three zones were necessary to categorise our participating farms: zone 7b (−15 to −12.2 °C), 9a (−6.7 to −3.9 °C) and 9b (−3.9 to −1.1 °C).

### Statistical data analysis and model building

Descriptive statistics (e.g. mean, range) were calculated for all data. STEC prevalence was estimated for the overall study and per livestock species. Generalised linear mixed models were used to assess the association between STEC presence in faecal samples and risk factors. The binary outcome of interest was whether each faecal sample was STEC-positive or negative. Univariate analysis assessed the distribution of variables. During bivariate analysis, variables with low variability, small cell sizes (<5) or large standard errors were either modified, collapsed if appropriate or discarded from model building. Correlation of numeric variables was measured with Spearman's rank correlation coefficient; those variables that were correlated 0.80 or more were highlighted during the model-building phase to evaluate for multicollinearity. To identify possible confounders, each variable was evaluated using a directed acyclic graph.

The glmer function was used from the lme4 package in R to build models, with farm added as a random effect to account for farm-level clustering effects [33]. Manual two-way stepwise variable selection was employed for model building. Variance inflation factors (VIF) identified possible multicollinearity and variables with a VIF over five were assessed for removal. Top models were compared and a final model was chosen based on the lowest Akaike Information Criterion (AIC) and smallest deviance, relative to the other models. Intraclass correlation (ICC) was calculated, which accounts for the proportion of the model variance explained by clustering and indicated whether a random effect was necessary. Diagnostic plots from the DHARMa package in R were used to assess the final model and included fitted *vs.* binned residuals, a Q-Q plot and the Kolmogorov–Smirnov test statistic [34]. Odds ratios and 95% confidence intervals (CI_95%_) were calculated for variables in the final model. All data analyses were performed using R Statistical Software version 1.4.1036^©^ [35].

## Results

### Study participants and farm characteristics

Sixteen farms participated in our study and were located in four regions of California: Central Valley (*n* = 10), Central Coast (*n* = 3), Shasta Cascade (*n* = 2) and the North Coast (*n* = 1). Four of the 16 farms (25.00%) raised livestock only, nine farms (56.25%) raised a combination of produce and livestock and used the animals to graze crop fields and three produce farms (18.75%) rented sheep seasonally to graze cover crops and did not raise any other animals. The rented sheep were present during the sampling day. Four farms were not organic (25.00%), 56.25% (9/16) were certified organic and 18.75% (3/16) used organic practices but were not certified by a third party.

### STEC prevalence

A total of 558 faecal samples were collected from 16 farms. Overall STEC prevalence (i.e. O157:H7 and non-O157 STEC) was 13.62% (76/558; CI_95%_ 10.88–16.75%). Farm-level STEC prevalence ranged from 0% to 30%; however, 37.50% (6/16) of farms had no positive samples. Of the 62.50% (10/16) of farms with positive STEC samples, the mean prevalence was 17.24%, with a median of 16.73%. Positive STEC samples were detected in all sampled livestock species. The count of each STEC O-serogroup and associated virulence genes per livestock species are shown in Table 1. Beef cattle had the highest STEC prevalence at 27.66% (13/47; CI_95%_ 15.62–42.64%) and second highest were dairy cattle at 18.18% (12/66; CI_95%_ 9.76–21.61%), with a 22.12% prevalence for all cattle combined. Goats had the next highest prevalence at 16.13% (15/93; CI_95%_ 9.32–25.20%), and then sheep 13.40% (28/209; CI_95%_ 9.09–18.78%). Swine samples had the lowest prevalence: 5.59% (8/143; CI_95%_ 2.45–10.73%). *Escherichia coli* O157:H7 was found only in cattle, with a prevalence of 5.31% (6/113). Of the 76 positive STEC faecal samples, 72 were non-O157 STEC, six were *E. coli* O157:H7 and one was untypeable. Three samples from beef cattle were positive for both *E. coli* O157:H7 and a non-O157 STEC. All six O157:H7 positive isolates were from one farm and contained virulence factors *stx2*, *ehxA*, *hlyA* and *eaeA*, but not *stx1*. O26 was the most prevalent O-serogroup, accounting for 27.85% (22/79) of positive isolates. *Stx2* was identified in 16.46% (13/79) of isolates, *stx1* was identified in 69.62% (55/79) and 13.92% (11/79) contained both *stx1* and *stx2*. In Table 1, isolates that shared the same virulence genes were from the same farm except the following, which originated from two farms each: O22 in dairy cattle, O103 in swine and O26 in swine. Additionally, O26 in goats was collected from three farms.

**Table 1.** Virulence genes discovered in 79 isolates from 76 positive Shiga toxin-producing *Escherichia coli* samples^a^ collected from 16 California diversified small-scale farms, between May 2015 and June 2016, characterised by livestock species, O-serogroup and virulence gene

Source	No. isolates^b^	O-serogroup	*ehxA*	*hlyA*	*eaeA*	*stx2*	*stx1*
Beef cattle	1	O109				+	
Beef cattle	1	O111	+	+	+	+	+
Beef cattle	1	O15				+	
Beef cattle	4	O157	+	+	+	+	
Beef cattle	2	O178	+	+		+	
Beef cattle	1	O22	+	+		+	+
Beef cattle	1	O26	+	+	+		+
Beef cattle	1	O46	+	+		+	+
Beef cattle	3	O5	+	+	+		+
Beef cattle	1	O7					+
Dairy cattle	1	O136	+	+		+	
Dairy cattle	2	O157	+	+	+	+	
Dairy cattle	2	O182	+	+	+		+
Dairy cattle	2	O22	+	+		+	+
Dairy cattle	1	O26	+	+			+
Dairy cattle	3	O43	+	+		+	+
Dairy cattle	1	O5	+	+	+		+
Goat	2	O176	+	+			+
Goat	11	O26	+	+	+		+
Goat	1	O43	+	+		+	+
Goat	1	NA	+	+	+		+
Sheep	7	O103	+	+	+		+
Sheep	2	O146	+	+		+	+
Sheep	3	O174					+
Sheep	5	O176	+	+			+
Sheep	7	O26	+	+	+		+
Sheep	4	O85	+	+			+
Swine	2	O100				+	
Swine	2	O103	+	+	+		+
Swine	1	O116		+	+		+
Swine	1	O165	+	+			+
Swine	2	O26	+	+	+		+

a One isolate was untypeable.

b Number of isolates containing the same O-serogroup and virulence genes.

### Risk factor analysis

Of the 16 participating farms, two were excluded from model building, due to incomplete questionnaires, leaving a total of 502 samples for model building. The mean, median and range of selected continuous variables assessed for inclusion during model building are shown in Table 2. Farms in this study ranged from two to 500 acres and had been farming 2–30 years. Stocking density rate was calculated by dividing the total number of livestock species, excluding poultry, by the total number of farm acres. Selected categorical variables, stratified by whether they were STEC-positive or negative, are presented in Table 3, and *P*-values were reported for *χ*^2^ test or Fisher's exact test if cell sizes were less than five. For instance, 28.99% (20/69) of positive samples came from farms that mixed livestock species within a barn, whilst only 15.70% (68/433) of negative samples came from farms with shared barns (*P*-value 0.012). Moreover, 72.46% (50/69) of positive samples were from farms that allowed livestock to have contact with bordering wild areas (e.g. streams, forest) (*P*-value 0.026).

**Table 2.** Mean, median and range (i.e. minimum and maximum) of selected continuous variables assessed for model building and collected during a cross-sectional study conducted from 2015 to 2016 on 14^a^ diversified small-scale farms in California

Description	Mean	Median	Minimum	Maximum
Number of farm acres	88.93	29	2	500
Average relative humidity (%)	52%	45%	23%	95%
Daily maximum temperature (°C)	28.32 °C	29.00 °C	11.70 °C	39.80 °C
Daily minimum temperature (°C)	10.85 °C	11.60 °C	0.40 °C	21.40 °C
Stocking rate (animals/acres)	3.11	1.48	0.20	13.75
Soil temperature (°C)	19.41 °C	20.60 °C	3.80 °C	28.70 °C
Total chickens per farm	1984	100	0	21 500
Total cattle (beef or dairy) per farm	8	0	0	47
Total goats (dairy or meat) per farm	8	5	0	31
Total sheep per farm	37	0	0	181
Total pigs per farm	36	10	0	325
Number of years farm had been in operation	10.29	7	2	30

a Two of the 16 participant surveys were not completed.

**Table 3.** Bivariate analysis for selected categorical variables stratified by positive (*n* = 69) or negative (*n* = 433) Shiga toxin-producing *Escherichia coli* status samples were collected during a cross-sectional study from 2015 to 2016 on 14^c^ diversified small-scale farms in California

Description	Variable category	STEC-negative ct (%)	STEC-positive ct (%)	*P*-value (*χ*^2^)
California regions based on USDA climate zones^a^	7b	87 (20.09%)	25 (36.23%)	0.003*
	9a	206 (47.58%)	20 (28.99%)	
	9b	140 (32.33%)	24 (34.78%)	
Different livestock species share the same barn	No	365 (84.30%)	49 (71.01%)	0.012*
	Yes	68 (15.70%)	20 (28.99%)	
Farm rotates different animal species within the same field	No	140 (32.33%)	27 (39.13%)	0.329
	Yes	293 (67.67%)	42 (60.87%)	
Livestock were allowed contact with wild areas	No	184 (42.49%)	19 (27.54%)	0.026*
	Yes	249 (57.51%)	50 (72.46%)	
Is the farm certified organic?^b^	No	129 (29.79%)	19 (27.54%)	0.073
	Not certified	125 (28.87%)	29 (42.03%)	
	Yes	179 (41.34%)	21 (30.43%)	
Does the farm raise swine?	No	82 (18.94%)	16 (23.19%)	0.507
	Yes	351 (81.06%)	53 (76.81%)	
Species of the collected sample	Cattle	88 (20.32%)	25 (36.23%)	0.002*
	Goats	78 (18.01%)	15 (21.74%)	
	Sheep	132 (30.48%)	21 (30.43%)	
	Swine	135 (31.18%)	8 (11.59%)	
Number of years in operation	6–30 years	270 (62.4%)	50 (72.5%)	0.137
	1–5 years	163 (37.6%)	19 (27.5%)	

a USDA zone information can be found online: https://planthardiness.ars.usda.gov. Three USDA zones were used to categorise participating farms: zone 7b (−15 to −12.2 °C), 9a (−6.7 to −3.9 °C) and 9b (−3.9 to −1.1 °C).

b Organic: some farms use organic practices, but were ‘not certified’ organic by a third party.

c Two of the 16 farm surveys were not completed.

* Indicates statistical significance with a P-value < 0.05.

### Final multivariable model results

The final mixed-effect multivariable logistic regression model is shown in Table 4 and had an AIC of 369.5, and a deviance of 351.5. The highest VIF for any variable in the model was only 2.02, which was below our threshold of five.

**Table 4.** Association between risk factors and the presence of Shiga toxin-producing *Escherichia coli* in faecal samples (*n* = 502) collected from 14^a^ diversified small-scale farms in California between May 2015 and June 2016, as demonstrated by a generalised linear mixed model, with farm as a random effect

Variable	Variable category	Estimate	OR	95% CI	*P*-value
Intercept		−2.57			0.001*
Daily maximum temperature °C	*numeric*	−0.06	0.95	0.91–0.98	0.003*
Sample source species	*Swine*	*Reference*			
	Goats	0.97	2.64	0.90–7.70	0.076
	Sheep	1.67	5.29	1.80–15.51	0.002*
	Cattle	1.53	4.61	1.64–12.96	0.004*
Multiple species shared a barn	*No*	*Reference*			
	Yes	1.83	6.23	1.84–21.15	0.003*
Livestock were allowed contact with wild areas	*No*	*Reference*			
	Yes	1.29	3.63	1.37–9.62	0.009*
Number of years in operation	*6–30 years*	*Reference*			
	1–5 years	−0.98	0.38	0.13–1.11	0.076

a Two of the 16 participant surveys were not completed.

*Indicates statistical significance with a *P*-value < 0.05.

Significant risk factors associated with the presence of STEC in the final model included: for every increase in the daily maximum temperature °C, the odds of a STEC-positive sample decreased (OR 0.95; CI_95%_ 0.91–0.98). The odds of a positive STEC sample were 4.61 times higher for cattle (OR 4.61; CI_95%_ 1.64–12.96) compared to swine and more than five times greater in sheep (OR 5.29; CI_95%_ 1.80–15.51). The odds of STEC increased by 6.23 times for those farms that housed multiple livestock species within the same barn *vs.* those farms that kept livestock separately (OR 6.23; CI_95%_ 1.84–21.15). The odds of a positive STEC sample were more than three times greater (OR 3.63; CI_95%_ 1.37–9.62) for a farm that allowed its livestock contact with wild areas. The effect of the number of years a farm had been in operation was not significant (*P*-value 0.076; CI_95%_ 0.13–1.11), but was included in the model as a possible confounder.

Diagnostically, the simulated residuals *vs.* predicted values did not show any significant problems: the Q-Q plot from DHARMa simulated scaled residuals was linear with no major deviations, and the Kolmogorov–Smirnov test indicated no deviation from uniform distribution of the scaled residuals [34]. The adjusted ICC was 0.08 for the final model, which signified the proportion of the variance that was explained by farm clustering and indicated the need for a farm random effect. The isSingular test function in the lme4 package was false, which indicated no singularities existed in the final model.

## Discussion

This is one of the first studies to describe the unique characteristics of DSSF in California while ascertaining significant associations between risk factors and the prevalence of STEC. This study detected STEC on more than half of the enrolled farms and typed O-serogroups including ones that cause serve illness in humans, including O157:H7, O26, O103 and O111 [23]. Significant risk factors for the presence of STEC included a negative association with the daily maximum temperature, whether multiple livestock species shared a barn, the host species of the collected faecal sample, and whether livestock had contact with wild areas.

Overall STEC prevalence measured for the 16 farms in this study was 13.62%. Six of the 16 farms had 0% STEC prevalence; however, due to the intermittent shedding of STEC which is affected by many factors, this result does not necessarily indicate that their livestock were not STEC reservoirs. Although STEC prevalence in livestock raised on large farms has been measured frequently in past studies, evaluation of STEC prevalence and associated risk factors estimated on DSSF is less common [28, 36–38]. However, a study conducted by USDA-APHIS collected faecal samples from dairy cows in 21 states and stratified *E. coli* O157:H7 prevalence between large dairies (i.e. 500 or more cows) and small dairies (i.e. 100 cows or less) and reported that small ranches had 29.4% *E. coli* O157:H7 and large dairies had 53.9% prevalence [39]. Although this report indicated that small farms have less *E. coli* O157:H7 than large farms, the 29.4% prevalence they detected on small dairies is still larger than the 18.18% we identified in dairy cattle. Risk factors for the transmission of foodborne pathogens on large farms may be different, especially if they only raise one crop or livestock type, instead of a diversity of species as seen on DSSF.

One of our previous studies measured a 4.17% STEC prevalence in sheep raised on a mixed crop–livestock organic farm in California, which was lower than the 13.4% prevalence in sheep identified in this study [40]. A 2005 California study that screened livestock at the California state fair, which usually hosts livestock raised on small-scale operations, observed a 3% prevalence of *E. coli* O157:H7 in pigs, but did not find O157:H7 in any other livestock samples including dairy cows, whereas our study identified O157:H7 in cattle but not pigs [41]. A 2002 study that also collected faecal samples at fairs in three states, identified an *E. coli* O157:H7 prevalence of 11.4% in cattle, 1.2% in swine and 3.6% in sheep and goats, whereas we measured a 5.31% (6/113) *E. coli* O157:H7 in all cattle (i.e. combined dairy and beef cattle samples) [42]. Animals shown at fairs are subjected to stress and comingling and may experience increased STEC shedding. Differing STEC prevalence in these aforementioned studies may reflect different management practices or other climate or animal-level factors, as reflected in our results. Additionally, since ruminants are the main reservoirs for STEC, our results indicating that STEC prevalence in swine is lower comparatively than the sampled ruminants are in agreement with previous research. However, pigs are still a livestock species of public health concern, as they harbour *E. coli* O157:H7 as indicated by many studies [36, 42,43]. Our model results also showed that cattle (OR 4.61) and sheep (OR 5.29) are significant factors in STEC presence. However, differences in location, laboratory methods and sampling methods make comparison between studies challenging.

More than half of the typed isolates in this study belonged to an O-group that is listed on the CDC list of the top 7 STEC of concern for public health, including six O157:H7, 22 O26, nine O103 and one O111 [23]. *Stx2* was identified in 16.46% (13/79) of isolates, and 13.92% (11/79) contained both *stx1* and *stx2*. Subtyping of *stx1* and *stx2* was not conducted in this study, therefore direct implications to public health due to virulence subtypes cannot be inferred. The *eaeA* gene, which allows STEC bacteria to attach to human host cells, was detected in 55.69% (44/79) of positive STEC samples, contrary to a study conducted by Dewsbury *et al*. (2015), which rarely reported *eaeA* in their non-O157 isolates from cattle faecal samples [44–47]. The *ehxA* gene, which is reported in severe human cases of STEC infection, was detected in 88.61% of the positive isolates (70/79) [46]. Compared to a study conducted by Djordjevic *et al*. (2004) in adult sheep and lambs, they detected *stx1*, *stx2* and *ehxA* in 78.2% of their positive isolates, *vs.* our study which only identified those three genes in 1.27% (1/79) of positive isolates. However, they reported 0.8% of their isolates had just *stx2* and *ehxA* genes, whereas, in this current study, 11.39% (9/79) of the positive isolates contained these two virulence genes [46]. The pathogenic STEC O-serogroups, genes and virulence factors identified in this study highlight the need for continued studies on DSSF, as well as the implementation of outreach efforts to stakeholders regarding pre-harvest food safety risks and the development of on-farm mitigation strategies.

Significant risk factors identified by the final mixed-effect model included daily maximum temperature °C, which ranged from 11.7 to 39.80 °C during the study period. An experiment that measured the decline of *E. coli* O157:H7 in inoculated manure at four temperatures, 7, 16, 23 and 33 °C, reported that *E. coli* O157:H7 declined significantly faster in manure at 23 and 33 °C than at 7 and 16 °C, for both oscillating and constant temperatures [48]. These study results confirm our model findings, which suggested that as the daily maximum temperature increased within our temperature range, the odds of finding STEC in a faecal sample were less likely. A study by Franklin *et al*. (2013) also identified daily maximum temperature as a significant risk factor, when conducting a study of STEC in wild ungulates in Colorado [49]. They detected a positive association between temperature and STEC presence in faecal samples, whereas our study identified a negative association with the daily maximum temperature [49]. However, the range of daily maximum temperatures in their analysis was narrower than our recorded temperatures, which may account for this difference. Although many studies indicated that higher STEC shedding occurs during summer months, California microclimates differ from each other and from the majority of seasons in other states [32]. California valleys and foothills experience low humidity and temperatures above 37.78 °C in the summer and autumn, which may affect STEC shedding [39]. For example, to compensate for the numerous microclimates in California in our study on *Campylobacter* spp., which included the same farms from this current study, we divided the California summer season into Coastal and Inland and the season was a significant risk factor in that final multilevel logistic regression model [11].

Differences in weather (i.e. humidity, temperature range) between states in the USA reveal a need to report the full range of measurements collected for studies estimating the effect of seasons on STEC shedding in livestock. For instance, a study that collected samples from conventional dairy and beef cattle in Michigan revealed that high average temperatures (more than 28.9 °C) measured 1–5 days before sampling had a 2.5 times greater odds of STEC than lower temperatures, which differs from our study results that suggested that STEC survival is less likely at higher maximum temperatures [50]. These Michigan results contradicted ours; however, the highest maximum daily temperature measured in our study (i.e. 39.8 °C) is not a temperature normally observed in many areas of the USA. The range of daily maximum temperatures for the Michigan study was 22.78–32.2 °C, with one 36.11 °C outlier. Additionally, our study included winter temperatures, while their study was only conducted in summer, and summers in Michigan include precipitation events, while little rainfall is observed during California summers. High temperatures or other weather discrepancies observed in different parts of the world may affect conclusions and interpretations of results.

Stanford *et al*. (2017) reported the effects of severe weather events on STEC shedding in Canadian cattle [51]. Although they also observed that STEC prevalence increased when ambient temperatures were higher than 28.9 °C, a separate finding indicated that the O-serogroup O26 had a significant reduction in prevalence during extreme heat in July and August [51]. Almost 28% of the O-serogroups in our study were O26, and the final model results may have been influenced by this strain. The survival of non-O157 STEC strains under different environmental conditions may account for variations in results between studies [51]. Moreover, physiological and behavioural changes in the host species during various temperature fluctuations or extreme weather events should also be considered [52]. For instance, Dawson *et al*. (2018) measured behavioural changes in cattle during increased temperatures, such as increased water consumption or change in grazing habits, as a possible driver of varying STEC prevalence [52]. Their simulation results indicated that higher summer temperatures may encourage more resting by cattle in crowded areas, such as under shade trees, which can lead to direct transmission of STEC. Since the aforementioned studies differ in conclusions regarding the direction of temperature on STEC shedding in livestock, this risk factor needs further investigation as perhaps there are underlying mechanisms accounting for the difference between results, including microclimates or animal-level factors [19, 51].

Our study also indicated that livestock sharing a barn with other animals resulted in 3.5 greater odds of a positive STEC sample. Multiple livestock housed in a barn could share pathogens by cross-contamination of food or water troughs or persistence of STEC in a barn environment that may not be subjected to regular cleaning [17, 53,54]. Other studies have indicated that STEC persists for long periods of time in barns or on surfaces within a farm environment. For instance, one study swabbed multiple barn surfaces at a dairy ranch and measured 14.9–19.1% STEC in samples from feeders and 11.3–18.0% on other surfaces [55]. Another study implicated water troughs as harbouring *E. coli* O157:H7 and inferred that shared water troughs play a role in the persistence and maintenance of continued *E. coli* O157:H7 infections in cattle [56]. A British study reported that housed beef cattle shed more STEC than unsheltered and suggested that this may be due to shared water sources or feeding bins or an accumulation of pathogens in a shared environment [53].

Finally, the final multivariable model included livestock in contact with wild areas, such as forests or wetlands, as a risk factor for the presence of STEC in faecal samples. Wildlife including feral pigs, deer and birds are known reservoirs of STEC [57–61]. A study conducted in California identified a low prevalence of *E. coli* O157:H7 in rodents (0.2%); however, they did not test for non-O157 STEC in samples, which may have a higher prevalence in rodents [62]. A 2016 published study discovered the *stx2* gene in over 20% of Canada geese faecal samples and 7% of nearby water samples from Lake Eric bordering Ohio, USA [58]. Another study of wild birds sampled on a California produce farm detected a low prevalence of 0.34% for O157:H7 and 0.5% for non-O157 STEC, which included O-serogroups O103 and O26 [61]. A case-control study conducted after 15 human cases of *E. coli* O157:H7 identified the source of STEC as those who ate fresh strawberries contaminated by deer faeces [60]. Livestock that graze in wild areas may be exposed to indirect sources of STEC, for instance through environmental contamination of soil or water, or because wildlife that live in these bordering wild areas enter agricultural areas and contaminate the pastures grazed by animals [24, 63].

Limitations of this study included a small sample size of farms and an inability to randomise sampled farms, so the model results are not generalisable to other regions and farms. Variables that should be included in future studies to increase our knowledge regarding carriage of foodborne pathogens on DSSF include the age of the animal and whether livestock have direct or indirect contact with neighbouring livestock.

## Conclusion

Many consumers perceive DSSF and outdoor-raised livestock as safer than food grown on large-scale conventional farms or meat animals raised in confinement systems. However, identification of STEC O-serogroups that are of public health concern indicates the need for mitigation strategies on DSSF, such as housing livestock species separately and restricting access to wild areas, in order to prevent the transmission of foodborne pathogens in a pre-harvest environment.

## Data Availability

The data that support the findings of this study are available from the corresponding author on reasonable request.
